# LC-HRMS-Based Non-Targeted Metabolomics for the Assessment of Honey Adulteration with Sugar Syrups: A Preliminary Study

**DOI:** 10.3390/metabo12100985

**Published:** 2022-10-18

**Authors:** Marianna Martinello, Roberto Stella, Alessandra Baggio, Giancarlo Biancotto, Franco Mutinelli

**Affiliations:** 1 National Reference Laboratory for Honey Bee Health, Istituto Zooprofilattico Sperimentale delle Venezie, 35020 Legnaro, Italy; 2 Department of Chemistry, Istituto Zooprofilattico Sperimentale delle Venezie, 35020 Legnaro, Italy

**Keywords:** fraud, honey adulteration, honeybee, LC-HRMS, metabolomics, sugar syrup

## Abstract

Honey is a natural product that is in great demand and has a relatively high price, thus making it one of the most common targets of economically motivated adulteration. Its adulteration can be obtained by adding cheaper honey or sugar syrups or by overfeeding honeybees with sugar syrups. Adulteration techniques are constantly evolving and advanced techniques and instruments are required for its detection. We used non-targeted metabolomics to underscore potential markers of honey adulteration with sugar syrups. The metabolomic profiles of unadulterated honeys and sugar beet, corn and wheat syrups were obtained using hydrophilic interaction liquid chromatography high-resolution mass spectrometry (LC-HRMS). The potential markers have been selected after data processing. Fortified honey (5%, 10% and 20%), honey obtained from overfeeding, and 58 commercial honeys were analyzed. One potential marker appeared with a specific signal for syrups and not for honey. This targeted analysis showed a linear trend in fortified honeys with a calculated limit of quantification around 5% of fortification.

## 1. Introduction

Honey is produced spontaneously by honeybees and is their own source of winter sustenance, a season in which they are unable to forage due to low external temperatures and scarce blooms [1]. Despite the potential ease of breeding bees, and therefore of obtaining products from the hive, honey is one of the foods most subject to adulteration and fraud [2,3]. In fact, over the years, honey has become an increasingly popular product, due to its unique taste and its known therapeutic properties, but also because of its increasing scarcity and price to produce [4] (pp. 309–334). The limiting factors responsible for the difficulties and risks in the entire sector are many, such as climate change and the persistence of the environmental impact of agricultural activity due to an incorrect use of phytosanitary drugs, diseases, pests, pathogens and aggressors of honeybees [5]. These factors can act synergistically with acute and chronic toxic effects with a consequent increased mortality of honeybees [6]. Other socio-economic aspects that put the beekeeping sector at risk are the increase in the average age of beekeepers worldwide, and the effects of globalization on the market, such as the marketing of low-cost honey and adulterated or even fake honey [4] (pp. 309–334). Despite all these problems, the worldwide consumption of honey has steadily increased over the past few decades. The increase in the world population, the preference for natural healthy foods and ready-to-serve products by a growing share of consumers, mean that the beekeeping sector is in constant growth, with the European Union (EU) being the second largest honey producer in the world after China [3,7].

Following the publication, in 2016, of a European control plan to determine the prevalence of fraud in honey, which highlighted the presence of foreign sugars in 14% (out of 2264) of the samples analyzed [8], the EU has identified honey as a food with a high risk of fraud [9] and the resolution of the European Parliament on 1 March 2018 called on the Commission to implement actions to combat honey-related fraud [10]. The control of fraudulent practices is the responsibility of the Member States, and in the respective national beekeeping programs for the three-year period 2020–2022, co-financed by the European Union, the importance of the fight against adulteration of honey was emphasized through more stringent controls and the favoring of the development and use of new analytical methodologies to search for adulteration [11].

Despite the apparent simplicity of the product, more than 180 components have been identified in honey, of which about 80% is represented by carbohydrates (mainly glucose and fructose) and 17% by water [12]. However, its numerous bioactive substances, such as phenolic compounds and flavonoids, organic acids, vitamins, enzymes, pigments and other natural antioxidant substances, make this product unique and contain therapeutic properties, which have been known since ancient times [13]. The high economic value of this product and the relative ease of adulteration by adding exogenous sugars, makes it one of the foods that, for several years, has been in the top positions in the rankings of foods most subject to economically-motivated fraud [2,3]. Fraud involving honey mainly includes the counterfeiting of the label to sell less valuable honey at higher prices, and adulteration, which is the dilution of honey by adding inferior quality honey or sugar syrups of vegetable origin such as corn, sugar cane, sugar beet, rice and wheat [14]. Sugar syrups can be incorporated into honey directly after production [15,16], or indirectly through the incorrect feeding of honeybees with complementary feeds during the main nectar flow period. In the latter case, honeybees store sugar syrup in brood combs and then move it to honey supers or store it directly in honey supers [17] (p. 190). Since sugars are naturally present in honey, the detection of adulterants is a challenge, and while the search techniques are constantly evolving, they fail to keep pace with the evolution of fraud [4] (pp. 309–334). In order to detect honey adulterants, many analytical techniques have been developed and extensively reviewed [18,19]. Among these techniques, thin-layer chromatography techniques, liquid and gas chromatography coupled with mass spectrometry (LC or GC-MS) and high-performance anion-exchange chromatography coupled with pulsed amperometric detection (HPAEC-PAD) have been described. Moreover, spectroscopy techniques, such as Raman, infrared spectroscopy and proton nuclear magnetic resonance (NMR), biosensors such as electronic-nose, e-tongue and optical fiber sensors have been applied, together with the stable carbon isotopic ratio analysis (SCIRA), which is currently the elective technique for the authentication of honey [20,21]. Recently, UV-VIS [22,23] and fluorescence spectroscopy [24,25] have also been used. It should be emphasized that these techniques are efficient when researching for known markers of sugar syrups. Alternatively, they require the use of very expensive instruments, as well as the integration of data with traditional analytical methods using chemometric techniques and experienced operators. In general, LC and GC based methods are suitable to perform the sugar profiling of honey, detecting oligosaccharides and polysaccharides derived from the addition of sugar syrups from corn and rice [26,27], and spectrometric techniques, including liquid chromatography combined with high resolution mass spectrometry (LC-HRMS) and LC-MS/MS, are used for marker detection. These targeted methods usually only detect one compound or a group of compounds at a time and are generally very reliable and sensitive. Therefore, they are preferred for determining adulteration when a primary marker of adulteration is present. Non-targeted analytical techniques, coupled with multivariate statistics (chemometry), including metabolomic studies, have become increasingly important in this field. Such techniques can detect unspecified signals, which can be compared to establish databases or to identify new markers of adulteration. As far as we know, few metabolomic studies have been carried out on honey to date. Most of them were aimed at determining the botanical origin of honey in terms of product authenticity [28,29,30]. However, Bachmann et al. (2022) [31] analyzed the metabolic profiles of syrups of different botanical origins with the NMR technique, identifying a marker for the detection of food fraud in each.

The aim of our work was to identify a single marker that was present in sugar syrups of different botanical origin, and that could be detected in adulterated honey at an acceptable sensitivity level. For this purpose, we used a non-targeted metabolomic approach, which is an ascending technique in food fraud research that can complement existing methodologies [32] by analyzing honey or syrup samples using LC-HRMS. The ultimate goal of our work was to identify markers of honey adulteration and then to transfer the analytical method by exploiting the most used targeted techniques that require less expensive and more widespread equipment in analytical laboratories.

## 2. Materials and Methods

### 2.1. Reagents, Chemicals, Honey and Syrup Samples

LC-MS grade water and acetonitrile (ACN) were from Fisher Scientific (Fisher Scientific, Milan, Italy). Acetic acid was obtained from Carlo Erba (Milan, Italy) and ammonium acetate was purchased from Merck (St. Louis, MA, USA). Isotope labeled internal standards (IS), namely, creatine-D_3_, leucine-5,5,5-D_3_, L-tryptophan-2,3,3-D_3_, and indole-2,4,5,6,7,3-acetic acid-D_5_, were purchased from Merck (St. Louis, MA, USA) and CDN Isotopes (Québec, Canada). Ten unadulterated honey samples of different botanical origin (multifloral, linden, acacia (*Robinia pseudoacacia*), alfalfa and “barena” (*Limonium narbonense*)), and 10 sugar syrups of different botanical origin (sugar beet, corn and wheat, see Table 1), marketed as complementary feed [33], were obtained from local retailers.

### 2.2. Sample Preparation

Samples were prepared by weighing 5 g of honey or syrup in a plastic tube and extraction was performed at a ratio of 10% (*w*/*v*) by adding a mixture of water and acetonitrile (80/20, *v*/*v*) containing IS at a final concentration of 0.5 ng µL^−1^. Samples were shaken for 15 min at room temperature and an aliquot of 2 mL was centrifuged at 18,000× *g* for 10 min at 20 °C. Finally, 0.5 mL was transferred to a glass vial for LC-HRMS analysis. A quality control (QC) sample was prepared by mixing an equal amount of each sample included in the study and subjecting it to extraction.

### 2.3. Liquid Chromatography and High-Resolution Tandem Mass Spectrometry (LC-HRMS)

Sample extracts were analyzed by an Ultimate 3000 ultra-high-pressure liquid chromatography (UHPLC) system coupled to a quadrupole-orbitrap (Q-Exactive) mass spectrometer (Thermo Fisher Scientific, San José, CA, USA). Analytes were separated in a 25 min elution gradient using water (A) and a mixture of ACN and water (90/10, *v*/*v*) (B), both containing 5 mM ammonium acetate and 5 mM acetic acid as mobile phases. The system was programmed to hold initial conditions (100% B) for 2 min and then the percentage of B was decreased linearly to 50% at 15 min. The column was washed at 50% of solvent B for 3 min and then the percentage of solvent B was increased to the initial conditions in 1 min. Finally, the column was equilibrated at initial conditions for 6 min. The autosampler was set at 20 °C, the sample injection volume was 1 μL, the hydrophilic interaction liquid chromatography column (Kinetex HILIC, 100 mm × 2.1 mm, 2.6 μm, Phenomenex, Torrance, CA, USA) was kept at 30 °C, and the flow rate was 300 μL min^−1^.

Non-targeted LC-HRMS metabolomics profiling was conducted in both positive and negative ionization modes using the following source parameters: Capillary temperature 325 °C, heater temperature 325 °C, sheath gas flow rate 35 a.u. (arbitrary units), auxiliary gas flow rate 10 a.u., S-lens voltage 50 V, and spray voltage 2.5 kV. The instrument was operated in full scan mode from 70 to 1000 *m/z* at a resolution of 70,000 full width at half maximum (FWHM) at 200 *m/z* using an automatic gain control (AGC) target of 3 × 10^6^ and an injection time (IT) of 250 ms.

### 2.4. Data Processing and Statistical Analysis

LC-HRMS data were acquired by randomizing the sample injection order during the non-targeted metabolomics analysis. After this, data were processed using Compound Discoverer software (Thermo Fisher Scientific, San José, CA, USA, version 2.1) to perform chromatographic peaks alignment, signals extraction and integration, and annotation. Processing parameters were the peak signal-to-noise ratio (S/N) of at least 1.5, maximum retention time shift of 0.5 min and *m/z* width of 5 ppm from 70 to 1000 *m/z*.

Then, *m/z* values from the mixture of labelled internal standards were included to check for the repeatability of chromatographic retention time and mass precision. After automatic processing by Compound Discoverer software, detected compounds were visually inspected and filtered. Only signals exhibiting a CV% value below 25% in the QC samples injected, every 4 samples in the analytical session (i.e., 6 QC injections in total), were considered as consistent and were thus included in the final data matrix [35]. Peak area values measured for each signal from the honey and syrup samples were then normalized to QC samples using a function embedded in the Compound Discoverer software. Thanks to this function, the peak area values of each detected compound in the honey and syrup samples were normalized using a regression curve built for each compound detected in all 6 injections of the QC sample. Normalized area values were finally exported to a spreadsheet for subsequent statistical evaluation.

Unsupervised Principal Component Analysis (PCA) was performed using SIMCA software (version 13.0, Umetrics, Umeå, Sweden) to visualize the general clustering, trends and outliers from relative quantification data from two datasets acquired in the positive and negative ionization polarities without creating a mathematical model for classification purposes. Data were mean-centered and Pareto-scaled before performing PCA. To evaluate the statistical significance of acquired signals, a volcano plot was built using a Microsoft Excel (Microsoft Corporation) spreadsheet to visualize the *p*-values resulting from the Student’s *T*-test and fold-change calculated as a ratio between syrup and honey samples. Potential markers of adulteration were selected among the detected compounds by applying two criteria: Area ratio between syrup and honey > 10 and *T*-test *p*-value < 0.001.

### 2.5. Targeted Analysis of Potential Markers of Honey Adulteration

Targeted experiments were conducted on a QC sample that was analyzed in full scan, combined with parallel reaction monitoring (PRM) mode using the same LC and source parameters described above to attempt to identify potential markers of adulteration. Full scan spectra were acquired in polarity switching (from 300 to 550 *m/z* and from 150 to 300 *m/z*, for positive and negative polarity, respectively) at a 70,000 resolution FWHM, as described above. Targeted MS/MS fragmentation spectra were acquired at a 35,000 resolution FWHM using an inclusion list containing an *m/z* value and predicted retention time of potential markers of adulteration. MS/MS fragmentation was carried out in a higher-energy collisional dissociation (HCD) cell using normalized collision energy (NCE) fixed at 23, AGC target of 2 × 10^5^, IT of 100 ms and an isolation window of 1.6 *m/z*.

After selection of the most promising marker of honey adulteration, a targeted PRM method for the selective fragmentation of this molecule was developed using the same chromatographic conditions described above. A calibration curve was built using unadulterated honey spiked with a mixture of 10 syrup samples of different botanical origins (each present in equal amount) at 5%, 10% or 20% (*w*/*w*). The calibration curve was used to study the linearity of the response, calculate a limit of quantification and analyze 58 honey samples that came from local producers to assess the applicability of the potential marker of adulteration. Quantification was performed using QuantBrowser software (Thermo Fisher Scientific, San José, CA, USA) by integrating the most intense precursor-to-product ion transition at the expected chromatographic retention time with an *m/z* tolerance of 5 ppm. Field samples obtained by overfeeding the honeybees with complementary feed (sugar beet syrup) were also processed. These samples were produced by administering a total of 7.5 kg of syrup per hive (divided into 2.5 kg packs) to two colonies in one week, and the honey was taken from honey supers the following week.

## 3. Results

### 3.1. Non-Targeted Metabolomics Fingerprint

**Figure 1:**
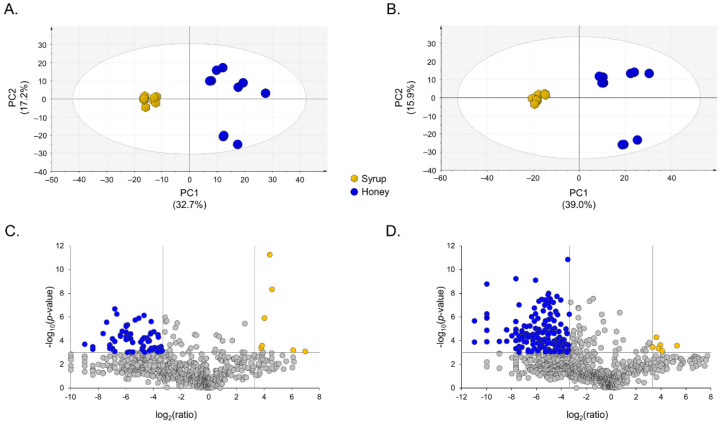
Principal component analysis (PCA) and volcano plots. PCA was performed to visualize the sample distribution and separation. Data were mean centered and scaled to unit variance before analysis. Metabolic profiles were acquired by applying HILIC in the positive ionization mode (**A**) and in the negative ionization mode (**B**). Honey samples and syrup samples were perfectly separated and clustered into different sides of each plot. Volcano plots were used to visualize the statistical significance and the fold change of the potential markers. Potential markers of adulteration passing the selected criteria (i.e., log_2_(syrup/honey) > 3.3 and −log_10_(*p*-value) < 3) were reported in yellow both for data acquired in the positive ionization mode (**C**) and in the negative ionization mode (**D**).

Aqueous extracts from honey and syrup samples were subjected to LC-HRMS analysis, together with a QC sample. We generated two data matrices, which came from the analysis conducted in positive or negative ionization polarity, which contained normalized area values for each detected compound (defined as a chromatographic peak characterized by a specific *m/z* value and a retention time). We detected 730 compounds in the positive ionization polarity and 954 compounds in the negative ionization polarity, which were consistently present in all the QC samples regularly injected along the analytical session (before, during and after) displaying a CV% below 25%. PCA, which was performed to compare the overall distribution and grouping of samples, demonstrated that all the QC samples were perfectly clustered together in the middle of the score plot, thus proving the absence of significant instrumental drift during data acquisition in both ionization polarities (Supplementary Figure S1). Then, data from the QC samples were removed from each data matrix and PCA was performed again on the honey and syrup samples. A clear separation was observed between honey and syrup samples, both in negative and positive ionization, regardless of the botanical origin (Figure 1A,B).

Potential markers of honey adulteration were screened by combining the fold change of the signal response for a given compound (i.e., ratio between syrup and honey > 10) and a *T*-test *p*-value cut-off (i.e., *p*-value < 0.001) to find the compounds that were highly abundant in syrup. Data were summarized in volcano plots (Figure 1C,D). Six compounds were detected in the negative ionization and seven compounds were detected in the positive ionization mode, which met the mentioned criteria (Table 2).

### 3.2. Targeted Analysis of Potential Markers

**Figure 2:**
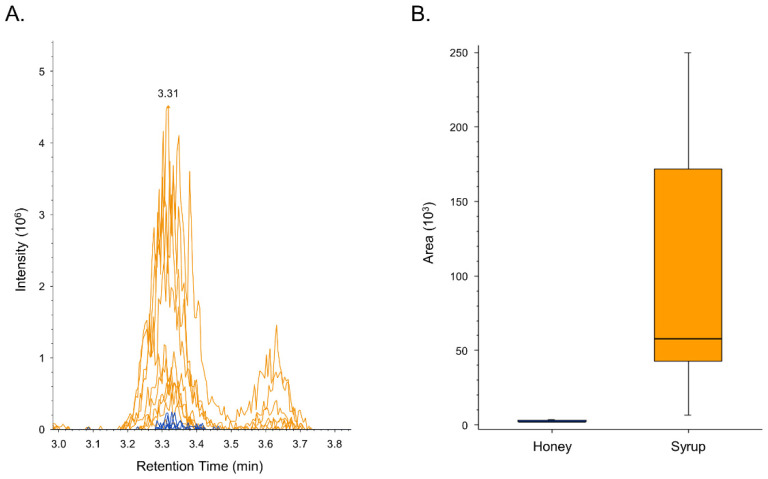
Extracted ion chromatogram of the potential marker of adulteration with *m/z* 515.1444. Signals coming from the syrup samples (yellow lines) and signal coming from the honey samples (blue lines) were overlaid for direct comparison (**A**). A box plot comparing normalized peak area values for the potential marker of adulteration shows that it was more abundant in syrup than in honey samples (**B**).

Potential markers of honey adulteration were further investigated by targeted experiments under the same instrumental conditions adopted for the non-targeted analysis by again extracting the same set of samples (i.e., 10 syrup and 10 honey samples of different botanical origin). Then, we exploited an analytical method by combining full scan acquisition and PRM in the positive and negative ionization polarities to obtain additional relative quantification values for the potential markers of adulteration and information about the fragmentation pattern of such compounds. Among the investigated compounds, we annotated two adducts of difructose dianhydride (*m/z* value of 363.06807 and 347.09408), an adduct of maltotriose (*m/z* value of 504.1916) and fuculose (*m/z* value of 243.0274). However, only one of the potential markers, *m/z* 515.1444, was selective for syrup samples (Figure 2A). The remaining signals were either not consistently detectable for all syrup samples, or were the adduct of other potential markers, or showed appreciable signals in one or more honey sample under investigation. Relative abundance values for the precursor ion of this potential marker in honey and syrup samples are summarized in a box plot (Figure 2B).

A tentative identification of this promising marker was completed on the basis of the accurate mass measured for the precursor ion and the MS/MS spectrum. We did not find product ions above *m/z* 515 and the isotopic distribution of the precursor ion indicated that it was a doubly charged one (Supplementary Figure S2). Additionally, we observed three other ions showing the same retention time and chromatographic profile. Such ions indicated that the one with *m/z* 515.1444 could potentially be a potassium adduct derived from a water loss (Supplementary Figure S3). Finally, a singly charged precursor ion, which we interpreted as the [2M+H]^+^ adduct, led us to suggest a [2M-H_2_O+H+K]^++^ adduct for the marker with an *m/z* value of 515.1444. Despite the presence of nine product ions for this molecule, some of which were doubly charged, we did not succeed in identifying the adulteration marker (Supplementary Figure S4). However, we proposed C_25_H_28_O_11_ as a potential molecular formula, which is supported by the observed product ions.

### 3.3. Quantification of the Most Promising Potential Marker

**Figure 3:**
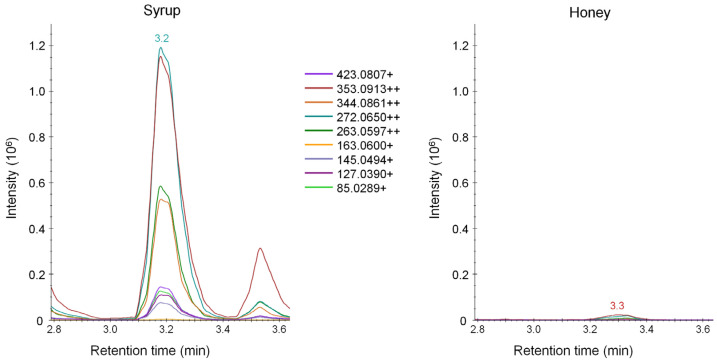
Parallel reaction monitoring (PRM) chromatograms of the most promising marker of honey adulteration. PRM chromatograms report nine precursor-to-product ion transitions derived from selective fragmentation of the precursor ion with *m/z* 515.1444 by applying a normalized collision energy of 23% in a representative syrup sample (left) and honey sample (right). The most intense precursor-to-product ion transition with *m/z* 272.0650 was exploited for targeted quantification purposes.

Finally, we verified the applicability of the potential marker of honey adulteration. To this end, we applied a targeted PRM method to selectively isolate and fragment the precursor ion of the most promising marker of adulteration (i.e., *m/z* 515.1444). We found that this molecule was specifically fragmented in nine product ions that were present in high abundance in the syrup samples but were very weak or totally absent in the honey samples (Figure 3).

After inspecting the fragmentation spectrum, the most intense precursor-to-product ion transition (i.e., *m/z* 272.0650) was exploited for targeted quantification purposes on additional honey samples.

We built a calibration curve using a mixture of 10 syrup samples of different botanical origin at 5%, 10% or 20% (*w/w*). The linearity of response was verified, given that the r^2^ value was found to be 0.994 ± 0.005 in four different analytical sessions. We established a LOQ value of 5% of adulteration, which was verified by analyzing a honey sample spiked at 5% (*w/w*) with a mixture of syrup in each analytical session. We used the LOQ value as a qualitative cut-off value to classify samples as non-adulterated or suspected of being adulterated with syrup.

### 3.4. Analysis of Honey Obtained from Sugar Beet Syrup Overfeeding and Commercial Honey

The most commonly adulterated proportion found on the market for acacia honey is estimated to be at least above 20% [36]. Therefore, we performed the analyses of honey samples based on a calibration curve starting from 5% of adulteration. We tested the targeted method to investigate 58 honey samples of different origins collected from local producers to look for signs of adulteration. Among these, for two honey samples (i.e., dandelion and acacia honey), we detected a signal corresponding to the marker of adulteration above the verified LOQ of the method, with an estimated syrup concentration of 5% and 20%, respectively, indicating the potential adulteration of such samples. For all the other honey samples, this compound was either detected well below the LOQ or not detected. Then, the method was tested on two honey samples obtained by experimentally overfeeding honeybees with syrup. Such samples showed a clear signal that corresponded to the metabolite marker of adulteration, giving an estimated concentration of 36% and 40%, respectively. Finally, the targeted analysis of 10 syrup samples confirmed the presence of the potential marker in the syrup of different botanical origin, such as sugar beet, corn and wheat, indicating its suitability for the assessment of adulteration in honey.

## 4. Discussion

The aim of the present research study was to develop a method to detect honey adulteration with the syrup of different botanical origin above a level of 5% (*w/w*). Recently, other studies were focused on the detection of honey adulteration [16,19], but the only official method for the detection of adulterants in honey is SCIRA, which requires very expensive instruments and highly qualified personnel. However, chromatographic techniques have proven to be reliable and sensitive towards adulteration markers, with the lowest detection limits at a level of 1% of adulteration using HPAEC-PAD [26]. LC has historically been used to determine the quality and authenticity of honey, and more recently, adulteration through targeted analyses by looking for specific markers or compounds at concentrations that indicate adulteration. Du et al. (2015) [27] used ultra-high performance liquid chromatography, coupled to quadrupole time-of-flight mass spectrometry (UPLC-QTOF-MS) for detecting adulteration of honey with corn syrup (evaluating the concentration of polysaccharides), high fructose corn syrup (detecting polysaccharides content and the specific marker DFA), rice syrup (by polysaccharides and the specific marker AFGP), invert sugar beet and sugar cane syrup (detecting DFA). In addition, a bee feeding experiment was conducted with the same syrups and an analysis of the resulting honey was performed. The results showed that this analytical method detects sugar syrups in honey down to 10% of addition. Additionally, for honey from bees fed syrups for three days, the same markers were present at approximately 60% of the concentration.

The present work was aimed at determining a single marker to detect the presence of sugar syrups of different botanical origins, such as corn, sugar beet and wheat, down to 5% of adulteration. This marker could be included in multiclass syrup screening by using chromatographic techniques with considerable savings in time and consumable materials.

The analytical strategy followed in our study was based on the comparison of the metabolomic fingerprint coming from a set of 10 unadulterated honey samples and from a set of 10 commercially available syrup samples (Table 1). LC-HRMS-based non-targeted metabolomics was conducted by combining HILIC and electrospray ionization, both in the positive and negative polarities in distinct analytical sessions. The majority of metabolites detected in negative and positive ionization polarities displayed a CV% below 25%, thus confirming the consistency of our analytical platform and the lack of instrumental drifts during sample analysis. The unsupervised PCA was performed on honey and syrup samples and showed a clear separation of honey from syrup samples, both in negative and positive ionization (Figure 1A,B), which indicated that syrup and honey samples have significant differences in their metabolomic fingerprints. Relative quantification data are summarized in volcano plots in Figure 1C,D and indicate that honey samples are characterized by a large number of metabolites, which are significantly higher in abundance with respect to the syrup samples (blue dots). However, a limited number of metabolites is significantly higher in abundance in syrup samples with respect to honey samples (yellow dots). The latter metabolites can be regarded as potential markers of adulteration, which are present in high amounts in the syrup samples of different botanical origins. Table 2 reports a list of potential metabolite markers that are characteristic of the syrup samples selected. According to the above-mentioned criteria, six compounds were detected in negative ionization and seven compounds were detected in the positive ionization mode.

These promising markers were then subjected to tentative identification by LC-HRMS/MS in order to elucidate the structure of such molecules. Among them, we found two adducts, which were annotated as difructose dianhydride, an adduct of maltotriose and fuculose. Difructose dianhydride was already described as a potential marker of honey adulteration with high fructose corn syrup, high fructose inulin syrup and inverted syrup [37,38], thus corroborating our findings. This marker was successfully used to develop a GC-MS method to detect honey adulteration down to 5% [37] and was also included in a LC-MS/MS method for the simultaneous detection of several adulterants in honey down to 10% [27]. Maltotriose is a trisaccharide composed of three glucose molecules. It is contained as traces in honey (mainly honeydew) [39], but it is considered an index of adulteration of honey when detected at high concentrations. It is the predominant sugar in rice syrup (52%) [40], but it is also present in high amounts in corn syrup (13%) [19]. Additionally, a large increase in malto-oligosaccharides, including maltrotriose, following honey adulteration with corn syrup down to 5% has been observed [12,41]. Finally, fuculose 1-phosphate aldolase is a biocatalyst that is widely used in the synthetic industry to produce rare sugars [42], however we did not find any references to the possible use of fucolose 1-phosphate as a marker of adulteration in honey.

In our study, only one of the investigated compounds, with *m/z* 515.1444, was shown to be highly selective for syrup samples, regardless their botanical origin. It displayed a very low signal in honey samples and a mean intensity that was approximately 70 times higher in syrup samples compared to the mean intensity in honey samples. During tentative identification, we observed a lack of product ions above *m/z* 515 and an isotopic distribution of the precursor ion, which is typical of a doubly charged ion, thus indicating that it could be a 2M adduct (Supplementary Figure S2). Additionally, we observed that ion with *m/z* 515.1444 could be derived from a water loss, as attested by a doubly charged ion (i.e., *m/z* 524.1484) showing the same retention time and chromatographic profile (Supplementary Figure S3). Moreover, we found two other doubly charged precursor ions with the same chromatographic retention time and profile (Supplementary Figure S3). Such ions showed an *m/z* of 507.1561 and 516.1614, displaying a mass difference of 7.99 *m/z* with respect to *m/z* 515.1444 and 524.1484, thus leading us to the conclusion that sodium was present in the former doubly charged adducts, and potassium in the latter ones. The presence of potassium adducts is supported by the high abundance of such mineral in honey [43]. Finally the presence of a weak, singly charged precursor ion with *m/z* 1009.3467, which could possibly be a [2M+H]^+^ adduct, suggested that the molecule with an *m/z* value of 515.1444 could be a [2M-H_2_O+H+K]^++^ adduct. Product ions generated by fragmenting the precursor ion with *m/z* 515.1444 supported our hypothesis, given that doubly charged product ions potentially contained potassium (Supplementary Figure S4). Based on the above-discussed MS data, a tentative formula can be indicated as C_25_H_28_O_11_ for this molecule (Table 2). While it was not possible to definitively identify or annotate such a promising marker, we submitted the proposed molecular formula (C_25_H_28_O_11_) to on-line databases (PubChem, Lipid Maps, HMDB). Among the results, we found several different flavonoids, some of which contained a sugar moiety.

One of the precursor-to-product ions was exploited to perform a targeted quantification of this marker of adulteration in 58 additional honey samples collected from local retailers and in two honey samples produced by experimentally overfeeding honeybees. The analysis of honey produced by overfeeding honeybees with syrup showed that such a marker was present well above the proposed cut-off level of 5%. Additionally, in two samples of honey collected from local producers, we found it above 5% level, thus confirming the potential reliability of the metabolite with *m/z* 515.1444 as a marker to detect adulteration in honey. Furthermore, the targeted quantification of this metabolite marker does not necessarily require HRMS instruments, given the specificity of its most intense precursor-to-product ion, thus increasing the possibility to screen for honey adulteration by routine laboratories working with conventional triple–quadrupole instruments.

## 5. Conclusions

The fight against food fraud is a pivotal aspect of food quality control, first to protect the interests of consumers, and second, to promote the proper development of the food industry. In particular, adulteration of honey affects not only the quality of the product, but also the confidence of consumers, and consequently the income of the beekeeping sector. A negative impact on honey producers could, in turn, cause damage to the entire ecosystem, as 80% of the world’s wild and cultivated plants are pollinated by pollinator insects and a decrease in the managed honeybee population would have negative ecological, agricultural and economic effects [5].

In this study, we adopted a LC-HRMS non-targeted metabolomics workflow to search for markers of honey adulteration. We found a metabolite marker that was present in a higher abundance (~70 times higher) in syrup samples, from sugar beet, corn and wheat, with respect to honey of different botanical origins. Using a targeted LC-HRMS/MS method based on PRM, we demonstrated the possibility to quantify such marker in honey contaminated down to 5% with syrup. The final aim was to validate the method for quantitative purposes, possibly transferring it to LC-MS/MS systems that are more commonly available in laboratories, such as the triple–quadrupole. To this end, future studies will be devoted to the identification of this metabolite and to the analysis of a larger cohort of honey and syrup samples from different botanical origins.

## Figures and Tables

**Table 1:** Mean composition and characteristics of honey and syrup samples investigated in the present study.

	Botanical Origin	Fructose (%)	Glucose (%)	Disaccharides (%)
honey ^1^		38	31	12
syrup 1	sugar beet	39	31	30
syrup 2	sugar beet	41–44	31–35	23–26
syrup 3	sugar beet and corn	42–46	23–28	13–19
syrup 4	sugar beet	43	32	25
syrup 5	sugar beet and corn	45	24	15
syrup 6	corn	42	n.d.	n.d.
syrup 7	wheat	32	26	n.d.
syrup 8	wheat	43–47	20–26	15
syrup 9	wheat	31–36	43–49	11–16
syrup 10	wheat	17–20	26–30	33–39

^1^ Average composition of the main carbohydrates present in honey (adapted from da Silva et al., 2016) [34].

**Table 2:** List of potential markers of honey adulteration in the non-targeted metabolomics experiment. For each metabolite, the observed *m/z* value, chromatographic retention time, ionization polarity, proposed adduct, calculated molecular formula, molecular weight, possible identification, ratio value (syrup/honey) and statistical significance are reported.

*m/z*	Retention Time (min)	Ion Mode	Adduct	Molecular Formula	Molecular Weight	Annotation	Ratio (Syrup/Honey)	*p*-Value
363.0681	3.31	positive	[M+K]^+^	C_12_H_20_O_10_	324.1057	bis-D-fructose 2′,1:2,1′-dianhydride	13.8	4.20 × 10^−4^
504.1916	2.55	positive	[M+NH_4_-H_2_O]^+^	C_18_H_32_O_16_	504.1690	maltotriose	14.6	2.78 × 10^−4^
347.0941	3.31	positive	[M+Na]^+^	C_12_H_20_O_10_	324.1057	bis-D-fructose 2′,1:2,1′-dianhydride	15.3	1.01 × 10^−3^
316.2114	1.27	positive	[M+H]^+^	-	315.2038	-	21.3	5.49 × 10^−12^
344.5875	2.55	positive	-	-	-	-	23.6	1.14 × 10^−3^
316.2114	1.05	positive	[M+H]^+^	-	315.2038	-	23.9	4.68 × 10^−9^
515.1444	3.31	positive	[2M-H_2_O+H+K]^++^	C_25_H_28_O_11_	504.1626	-	69.5	6.65 × 10^−4^
243.0274	1.23	negative	[M-H]^−^	C_6_H_13_O_8_P	244.0348	fuculose 1-phosphate	10.1	3.52 × 10^−4^
270.9254	7.16	negative	-	-	-	-	12.5	5.18 × 10^−5^
245.0244	1.24	negative	[M-H]^−^	C_8_H_12_N_2_O_3_P_2_	246.0316	-	14.1	4.60 × 10^−4^
270.0468	1.24	negative	[M-H]^−^	C_7_H_13_NO_10_	271.0540	-	15.5	2.44 × 10^−4^
194.0298	0.93	negative	[M-H]^−^	C_5_H_9_NO_7_	195.0371	-	17.2	7.47 × 10^−4^
183.0290	1.64	negative	-	-	-	-	39.2	2.62 × 10^-4^

## Data Availability

Data will be made available upon request from the corresponding author.

## Supplementary Materials

The following supporting information can be downloaded at: https://www.mdpi.com/article/10.3390/metabo12100985/s1, Figure S1: Principal component analysis (PCA) performed on data acquired during the non-targeted metabolomics analysis to assess acceptability of the analytical sequences; Figure S2: Full MS scan and MS/MS fragmentation spectra of ion with *m/z* 515.1444; Figure S3: Extracted ion chromatograms of five adducts proposed for the most promising marker of honey adulteration; Figure S4: Tentative marker identification.
