# Supplementary material for: LC-HRMS-Based Non-Targeted Metabolomics for the Assessment of Honey Adulteration with Sugar Syrups: A Preliminary Study
Source: Metabolites. 2022 Oct 18;12(10):985. doi: 10.3390/metabo12100985 (PMC9607529; doi:10.3390/metabo12100985)

Supplementary Figure S1. Principal component analysis (PCA) performed on data acquired during the non-targeted metabolomics analysis to assess acceptability of the analytical sequences. Metabolic profiles were acquired applying different chromatographic and ionization conditions: HILIC in positive ionization mode (A), HILIC in negative ionization mode (B). Quality Control (QC) samples (red pentagons) are clustered together in the middle of each plot, indicating the stability and repeatability of signals measured in the analytical sequences. Honey samples are represented by blue dots, while syrup samples are represented by yellow hexagons.

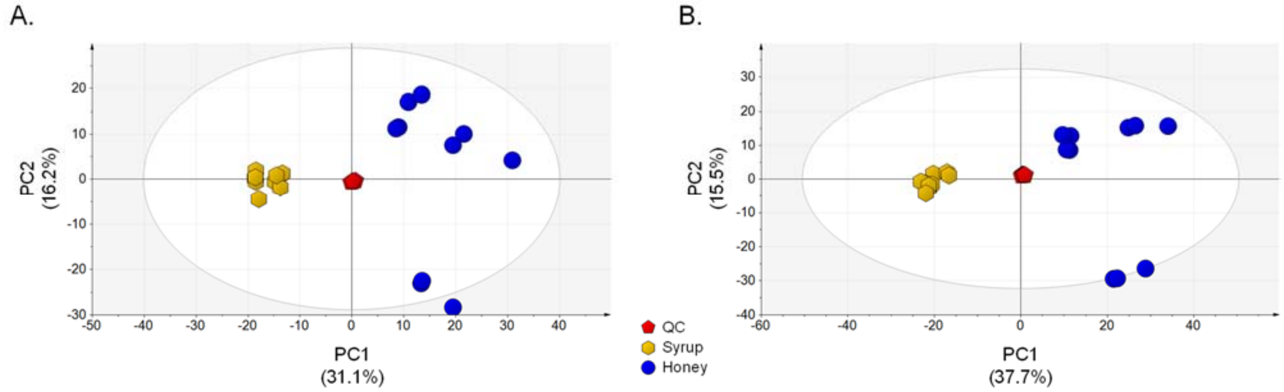

Supplement: Supplementary file 1 [file metabolites-12-00985-s001.zip › Supplementary Figure S1.pdf]
