# Supplementary material for: LC-HRMS-Based Non-Targeted Metabolomics for the Assessment of Honey Adulteration with Sugar Syrups: A Preliminary Study
Source: Metabolites. 2022 Oct 18;12(10):985. doi: 10.3390/metabo12100985 (PMC9607529; doi:10.3390/metabo12100985)

Supplementary Figure S2. Full MS scan and MS/MS fragmentation spectra of ion with *m/z* 515.1444. This figure reports a full scan spectrum (A) acquired by analysing a representative extract of a syrup sample in positive ionization mode using HILIC separation, and the corresponding HCD fragmentation spectra (B and C) obtained by applying a normalized collision energy of 23%. The isotopic distribution of the precursor ion suggested the presence of two charges (A). We observed the presence of nine precursor-to-product ion transitions deriving from this doubly charged ion (B). The absence of relevant product ions above *m/z* 515 indicated that such doubly charged ion was probably deriving from a $2M^{++}$ instead of an M^{++} adduct (C).

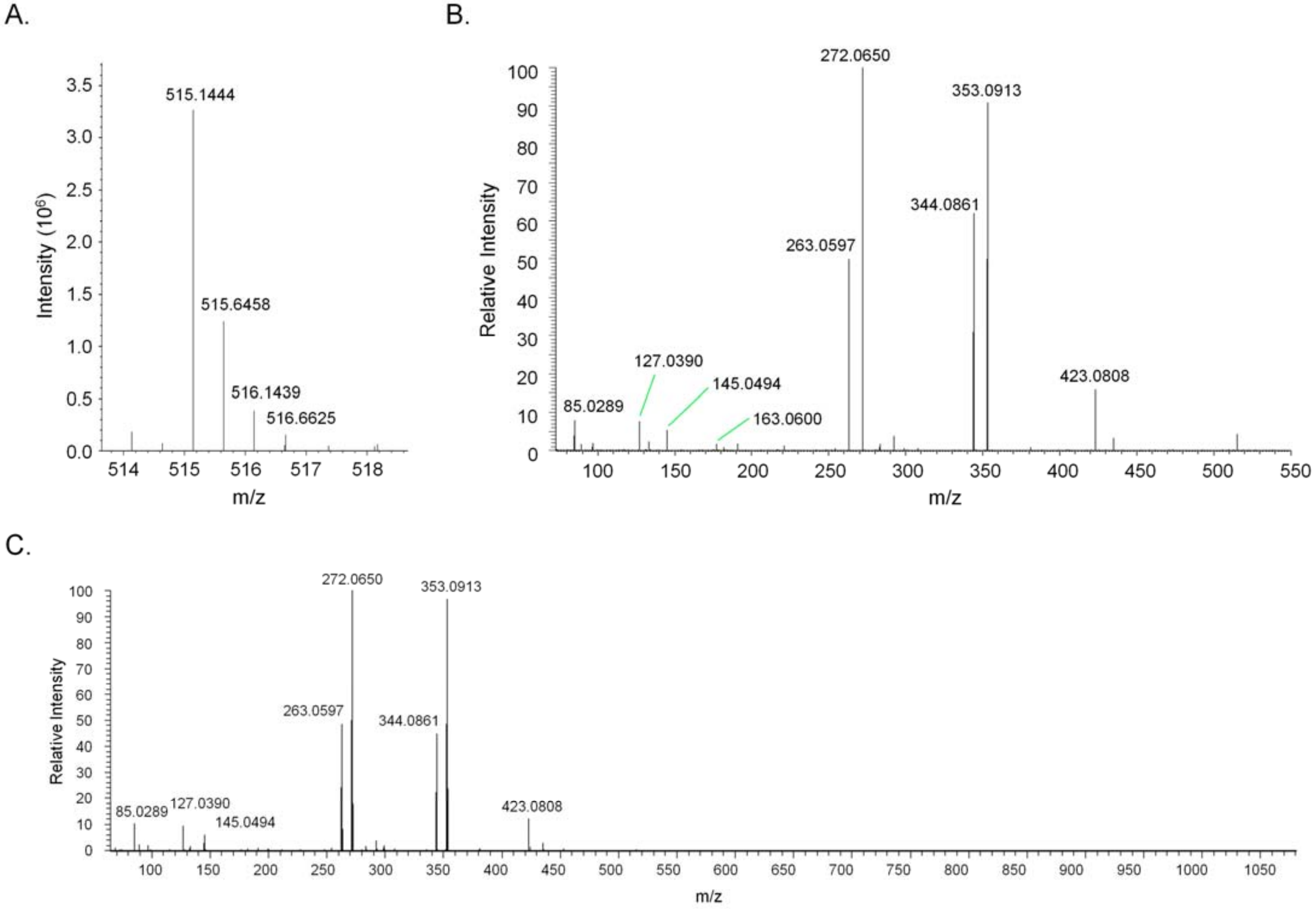

Supplement: Supplementary file 1 [file metabolites-12-00985-s001.zip › Supplementary Figure S2.pdf]
