# Supplementary material for: LC-HRMS-Based Non-Targeted Metabolomics for the Assessment of Honey Adulteration with Sugar Syrups: A Preliminary Study
Source: Metabolites. 2022 Oct 18;12(10):985. doi: 10.3390/metabo12100985 (PMC9607529; doi:10.3390/metabo12100985)

Supplementary Figure S3. Extracted ion chromatograms of five adducts proposed for the most promising marker of honey adulteration. Five different adducts for this molecule have been generated starting from the inferred molecular formula of $C_{25}H_{28}O_{11}$. The resulting signals together with the corresponding isotopic distribution have been extracted from full scan spectra acquired analysing a representative syrup sample. All these signals display the same chromatographic retention time, a mass uncertainty (Δ ppm) below 5 ppm with respect to the theoretical value, supporting our hypothesis of the presence of a $[2M-H_2O+H+K]^{++}$ adduct for the marker with *m/z* 515.1444.

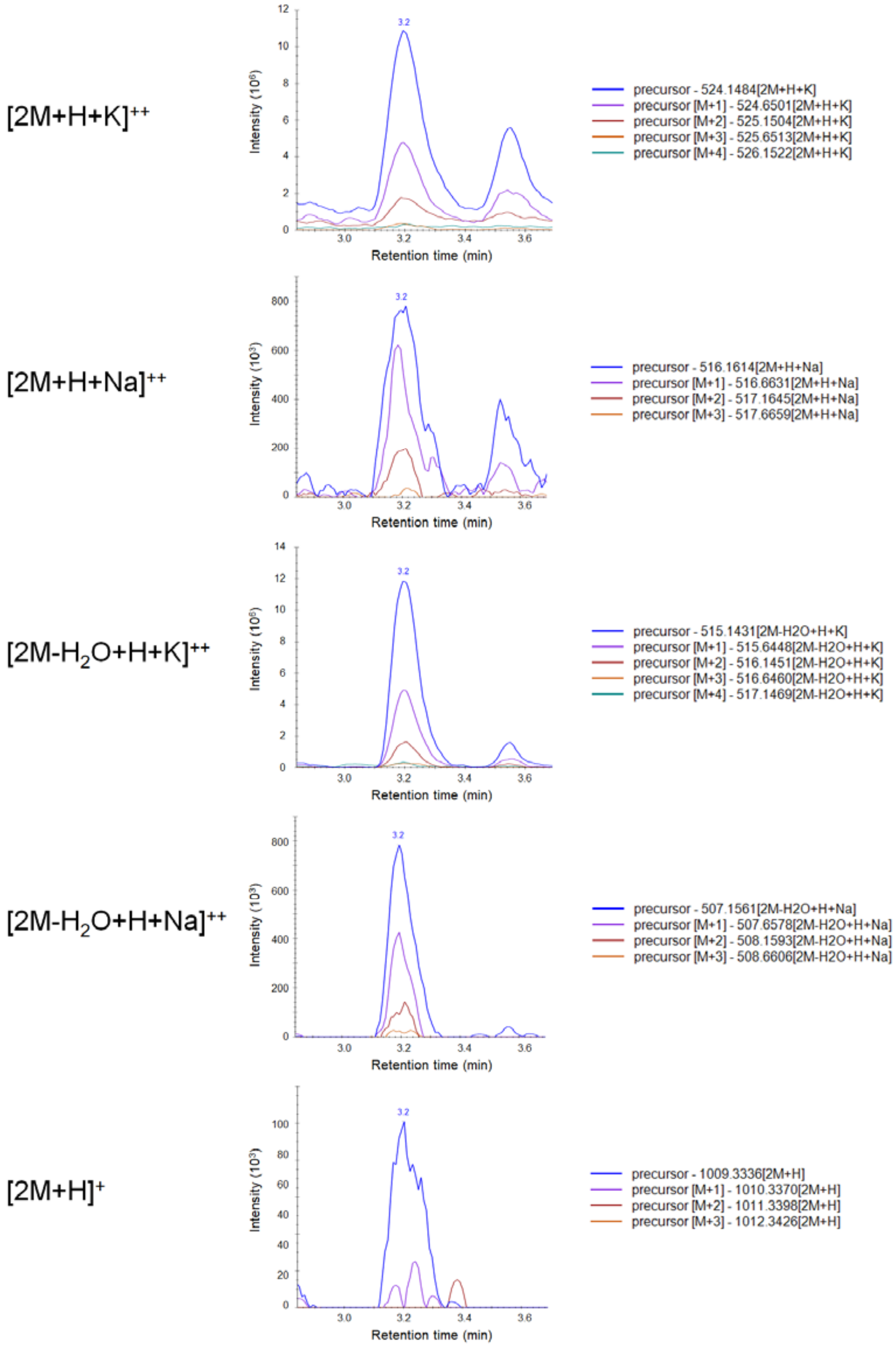

Supplement: Supplementary file 1 [file metabolites-12-00985-s001.zip › Supplementary Figure S3.pdf]
