# Supplementary material for: LC-HRMS-Based Non-Targeted Metabolomics for the Assessment of Honey Adulteration with Sugar Syrups: A Preliminary Study
Source: Metabolites. 2022 Oct 18;12(10):985. doi: 10.3390/metabo12100985 (PMC9607529; doi:10.3390/metabo12100985)
Supplement: Supplementary file 1 [file metabolites-12-00985-s001.zip › Supplementary Figure S4.pdf]

Supplementary Figure S4. Tentative marker identification. MS/MS spectra acquired for marker ion with *m/z* 515.1444 obtained by analysing a representative extract of a syrup sample in positive ionization mode and applying HCD fragmentation with normalized collision energy of 23%. It was possible to inspect the isotopic distribution of five out of nine product ions, which indicated that four of them were doubly charged product ions (++) and one of them was a singly charged product ion (+), as reported above each inset of the fragmentation spectrum (A).

The lower panel (B) shows the corresponding HCD fragmentation spectrum in which we reported a potential molecular formula together with mass uncertainty with respect to the theoretical value (Δ ppm) for the observed product ions. The left part of the spectrum displays three ions (i.e. *m/z* 127.0390, 145.0494, 163.0600) characterized by a series of water losses (i.e. mass difference of 18.011 *m/z*). On the right part of the spectrum, we proposed a potassium adduct for the singly charged ion with *m/z* 423.0808 given that potassium was present in the precursor ion. In the central part of the spectrum, we observed four doubly charged ions. We proposed a doubly charged adduct for ion with *m/z* 272.0650 (i.e. adduct $[M+H+K]^{++}$) and its doubly charged water loss (i.e. mass difference of 9.005 *m/z*) for ion with *m/z* 263.0597 (i.e. adduct $[M-H_2O+H+K]^{++}$).

Finally, we tentatively inferred similar adducts for ions with *m/z* 344.0861 and 353.0913. One of the possible calculated adducts for ion with *m/z* 344.0861 is $[C_{31}H_{36}O_{15}+H+K]^{++}$ that could be interpreted as $[C_{25}H_{28}O_{11}+C_6H_8O_4+H+K]^{++}$, thus representing a potassium adduct of M associated to another of the observed singly charged product ions (i.e. *m/z* 145.0494). In a similar way, one of the possible calculated adducts for ion with *m/z* 353.0861 is $[C_{31}H_{38}O_{16}+H+K]^{++}$ that could be interpreted as $[C_{25}H_{28}O_{11}+C_6H_{10}O_5+H+K]^{++}$, thus representing a potassium adduct of M associated to another of the observed singly charged product ions (i.e. *m/z* 163.0600).

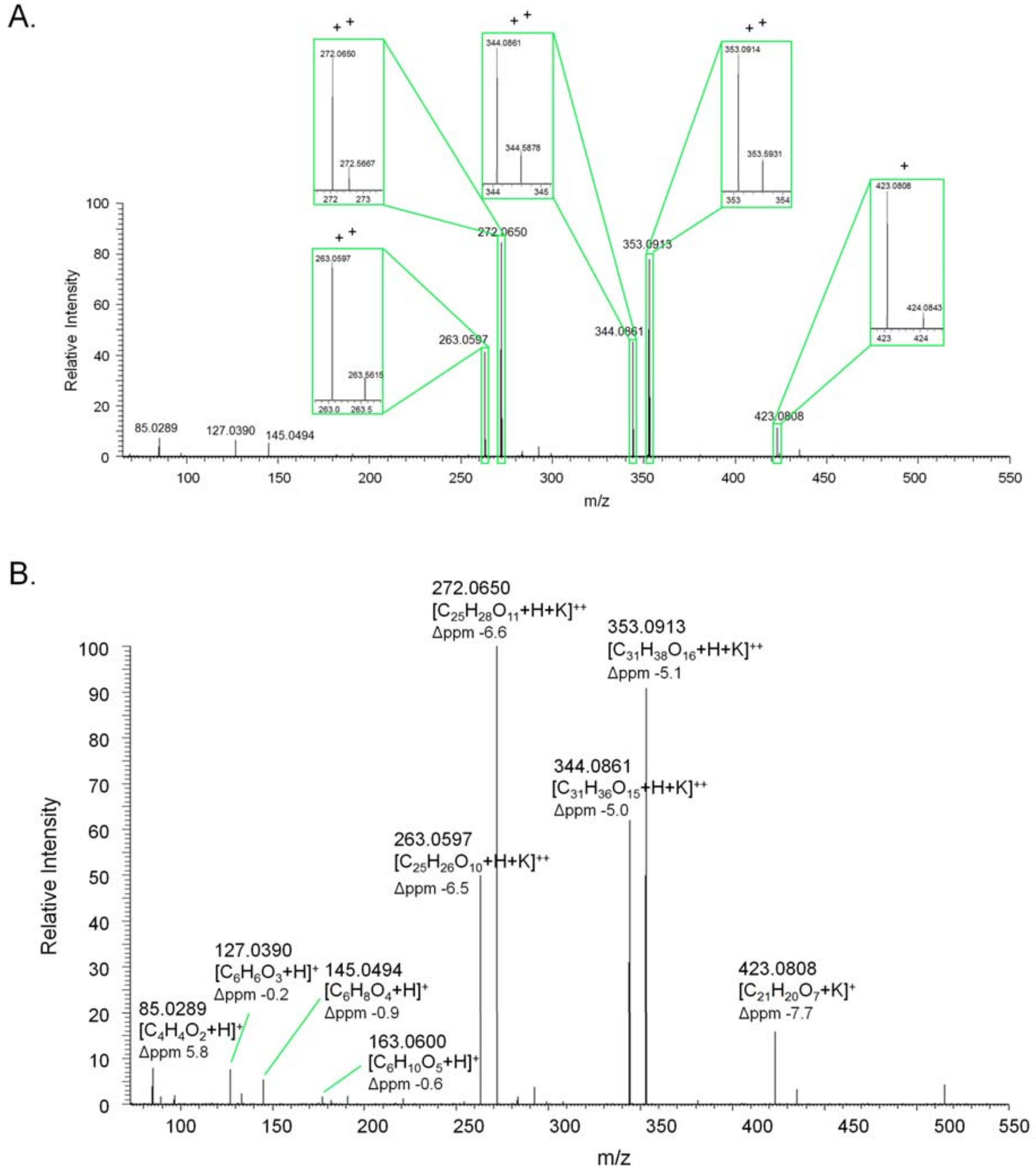
A.
Relative Intensity
m/z
85.0289
127.0390
145.0494
263.0597
272.0650
344.0861
353.0913
423.0808
272.5667
344.5878
353.5931
263.5615
424.0843
B.
272.0650
[C25H28O11+H+K]++
Δppm -6.6
353.0913
[C31H38O16+H+K]++
Δppm -5.1
344.0861
[C31H36O15+H+K]++
Δppm -5.0
263.0597
[C25H26O10+H+K]++
Δppm -6.5
127.0390
[C6H6O3+H]+
Δppm -0.2
145.0494
[C6H8O4+H]+
Δppm -0.9
85.0289
[C4H4O2+H]+
Δppm 5.8
163.0600
[C6H10O5+H]+
Δppm -0.6
423.0808
[C21H20O7+K]+
Δppm -7.7
Relative Intensity
m/z
